# Diabetic Ketoacidosis-Induced “Terrible Triad” Associated With Seizures and Acute Renal Failure: A Report of a Rare Case

**DOI:** 10.7759/cureus.45214

**Published:** 2023-09-14

**Authors:** Shrenil Kavathia, Sharvil Kataria, Nirav Patel, Sagar Patel

**Affiliations:** 1 Internal Medicine, Shri Jalaram Arogya Seva Trust Hospital, Meghraj, IND; 2 Internal Medicine, Siddhi Heart and Medical Hospital, Ahmedabad, IND; 3 Neonatology, Orange Neonatal and Pediatric Hospital, Ahmedabad, IND

**Keywords:** seizures, terrible triad of endocrinology, acute pancreatitis, diabetic ketoacidosis, hypertriglyceridemia, diabetes mellitus

## Abstract

We report a case of a 17-year-old male patient who came to the emergency department with abdominal pain, headaches for two days, lethargy, and Kussmaul breathing. Diabetic ketoacidosis (DKA) was diagnosed. The patient’s clinical course was complicated with a severely elevated triglyceride (TG) level (25,585 mg/dL), acute pancreatitis, renal involvement, and generalized seizures. The proposed mechanism is triglyceride excess due to increased lipolysis, resulting in the formation of excess free fatty acids. The objective of this case report is to present and describe the clinical features, laboratory investigations, case management, and natural course of hypertriglyceridemia in DKA.

## Introduction

An acute metabolic complication known as diabetic ketoacidosis (DKA) primarily affects people with type 1 diabetes mellitus [1]. Type 1 diabetes mellitus results from the destruction of insulin-producing β-cells in the pancreatic islets of Langerhans [2].

DKA is the result of absolute or relative insulin deficiency combined with counter-regulatory hormone excess (glucagon, catecholamines, cortisol, and growth hormone). The decreased ratio of insulin to glucagon promotes gluconeogenesis, glycogenolysis, and ketone body formation in the liver [3]. In DKA, insulin deficiency activates lipolysis in adipose tissue, releasing increased free fatty acids, which accelerates the formation of very low-density lipoprotein (VLDL) in the liver. Additionally, decreased removal of VLDL from the plasma by peripheral tissue lipoprotein lipase results in hypertriglyceridemia [4]. DKA-induced hypertriglyceridemia is a rare cause of acute pancreatitis, which accounts for around 4% of cases [5]. This case involves DKA-induced severe hypertriglyceridemia complicated by acute pancreatitis, acute renal involvement, and generalized seizures.

## Case presentation

**Figure 1:**
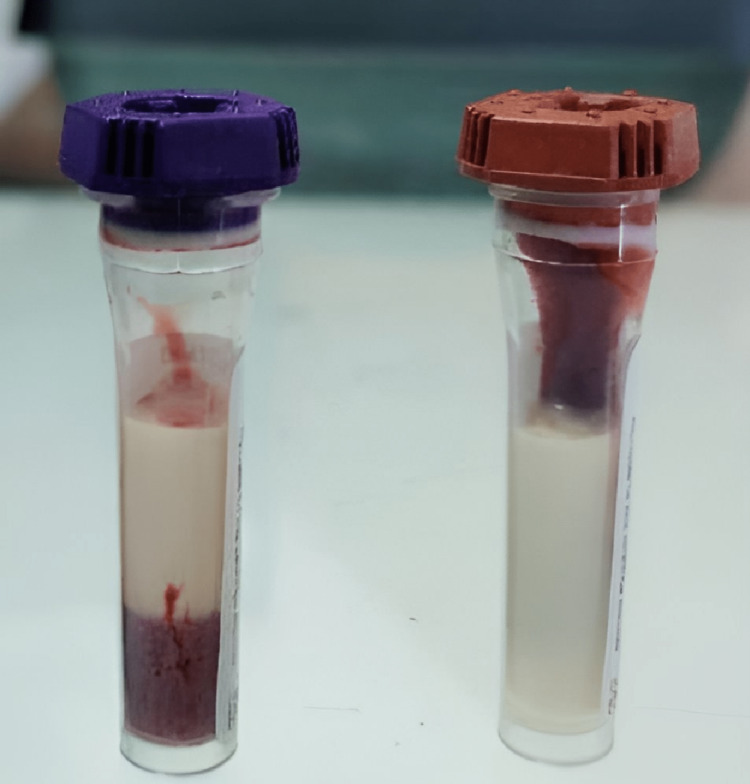
Hyperlipidemic blood sample on the day of admission

A 17-year-old male presented to the hospital in the western part of India with abdominal pain, headaches for two days, lethargy, and Kussmaul breathing for the last few hours. The patient was admitted to the intensive care unit (ICU) for lethargy and severe respiratory distress. The patient was put on ventilator support. Fingerstick blood glucose level was greater than 500 mg/dL, and initial arterial blood gas analysis (ABGA) resulted in a pH of 6.9, partial pressure of carbon dioxide (PaCO_2_) of 21 mmHg, partial pressure of oxygen (PaO_2_) of 93 mmHg, and bicarbonate (HCO_3_-) of 4 mM/L, which was suggestive of severe metabolic acidosis; hence, a probable diagnosis of DKA was made. His Glasgow Coma Scale score was 4 (E1M2V1), so he was immediately intubated, ventilation was commenced, and management of shock was started. As per the British Society For Paediatric Endocrinology and Diabetes (BSPED) guidelines for DKA 2020, aggressive intravenous (IV) fluids and injection insulin were started after collecting blood samples. Normal saline (NS) bolus of 10 mL/kg was administered, along with bicarbonate correction. IV antibiotics were started for suspected sepsis. After the initial bolus of NS 20 mL/kg, the patient was started on noradrenaline for low diastolic blood pressure. Serum amylase was ordered because of complaints of abdominal pain, which came back 394 U/L, which implied that the patient had acute pancreatitis. The blood investigation also reported leukocytosis with a triglyceride (TG) level of 25,585 mg/dL, serum glutamic pyruvic transferase (SGPT) of 70.4 U/L, creatinine of 1.8 mg/dL, and C-reactive protein (CRP) of 42.9 mg/dL. A picture of the blood-containing vacutainer on the day of admission is shown in Figure 1, and it shows a milky supernatant due to the elevated TG levels.

The patient developed generalized seizures; hence, fosphenytoin was administered (20 mg/kg/dose). However, he had persistent seizures, so a loading dose of levetiracetam (40 mg/kg/dose) was dispensed. A neurologist was consulted for the same, who advised a brain magnetic resonance imaging (MRI), which revealed a small acute non-hemorrhagic infarct in the right half of the midbrain without mass effect or midline shift. The electroencephalogram (EEG) report showed abnormal diffuse, slow background activity suggestive of encephalopathy. The patient was continued on fosphenytoin (6 mg/kg/day) and levetiracetam (40 mg/kg/day) maintenance doses. The laboratory parameters were monitored throughout admission (Table 1).

**Table 1:** Sequential laboratory parameters PaCO_2_: partial pressure of carbon dioxide, PaO_2_: partial pressure of oxygen, HCO_3_-: bicarbonate

Parameters	Reference values	Initial	After 7 hours	After 12 hours	Day 2	Day 3	Day 4	Day 5	Day 6	Day 7	Day 14	Day 21
Serum triglycerides (mg/dL)	<150	25,585	-	-	16,600	9,500	7,500	-	-	135	-	-
Total cholesterol (mg/dL)	<200	1,277	-	-	1,250	-	844	-	-	-	-	-
Serum urea (mg/dL)	5-20	28.3	-	-	-	149	119	159	215	195	73.5	-
Serum creatinine (mg/dL)	0.6-1.2	1.8	-	-	-	4	4.3	5.1	5.8	6.22	1.25	-
Serum amylase (U/L)	40-120	394	-	-	-	-	93	-	-	-	-	-
pH	7.35-7.45	6.9	7.06	7.1	7.2	7.27	7.28	7.44	7.47	-	-	-
PaCO_2 _(mmHg)	38-42	21	21	17	21	16	14	17	28	-	-	-
PaO_2 _(mmHg)	75-100	93	128	128	138	127	112	138	113	-	-	-
HCO_3_^- ^(mEq/L)	22-28	4	6.5	5.4	8.6	7.6	6.6	15.5	19.9	-	-	-
Total leukocyte count (/mm^3^)	4,000-11,000	19,120	-	-	-	6,270	4,230	-	-	9,210	10,430	7,960
Hemoglobin (g/dL)	13.3-16.6	10.7	-	-	-	9	9.2	-	-	9.2	7.3	8.2
Na^+ ^(mEq/dL)	135-145	126	-	-	139	146	155	151	152	152	147	-
K^+ ^(mEq/dL)	3.6-5.2	7.5	-	-	3.6	3	4.3	4.2	4.5	4.6	2.9	-
Serum acetone (mmol/L)	<0.6	72	-	-	-	5	-	-	-	-	-	-

On the third day of hospitalization, a nephrologist was consulted due to bilateral mild renal involvement seen on abdominal ultrasonography (USG) and for rising serum creatinine and urea levels. A hemodialysis catheter was inserted, and hemodialysis was initiated and continued for four cycles over a week. The TG level was on a downward trend, and the leukocyte count returned to normal. On the fifth day of hospitalization, the ABGA revealed a normal pH and normal PaO_2_ and PaCO_2_.

On the seventh day of hospitalization, the patient was weaned from the ventilator and kept on low-flow oxygen for the next four days. The TG level had returned to a normal level. Physiotherapy was started during the hospital stay. After three days, feeding via Ryles tube was started and was gradually upgraded. The blood culture remained sterile after five days of incubation. An endocrinologist was consulted, and the patient was shifted to a subcutaneous regular insulin injection. Repeat blood investigations showed improvement in serum creatinine levels. The patient developed melena episodes, so a unit of packed red cells was transfused. The patient was tolerating the management well, so physiotherapy was stopped, and he was discharged after 21 days of admission. The patient was diagnosed with diabetes mellitus type 1 as a result. The patient and his parents were taught how to administer insulin using an insulin pen injection.

## Discussion

This case report describes a patient presenting with symptoms consistent with diabetic ketoacidosis and acute pancreatitis. Blood reports confirmed hypertriglyceridemia and severe metabolic acidosis. Milky blood, as described by Chaurasiya et al. [6], is a clear supernatant layer of triglycerides above the cellular components of blood; however, the patient did not have any cutaneous manifestations of hypertriglyceridemia. The patient’s clinical course was complicated by generalized seizures, an MRI suggestive of a small acute non-hemorrhagic infarct in the right half of the midbrain without mass effect or midline shift, and a USG suggestive of bilateral mild renal involvement. In this patient, hypertriglyceridemia was well controlled with insulin without lipid-lowering agents. However, in severe hypertriglyceridemia, Furuya et al. [7] recommended plasmapheresis to avoid severe adverse effects.

Diabetic ketoacidosis is an acute and severe complication of type 1 diabetes mellitus. DKA can be accompanied or complicated by the presence of hypertriglyceridemia and pancreatitis. This is known as the “terrible triad” or “enigmatic triad,” which can be an unusual presentation of type 1 and type 2 diabetes mellitus [8]. The actual trigger is not known, but after onset, it creates a “domino effect” [9]. The development of DKA from diabetes mellitus can lead to a continuous loop of events. DKA leads to hypertriglyceridemia due to lipolysis promoted by an insulin-deficient state, which can be complicated by acute pancreatitis [10]. This is one of the mechanisms of the terrible triad formation. In addition to this, pancreatitis leads to inflammation, fibrosis, and sclerosis of pancreatic tissue, which leads to the exacerbation of diabetes mellitus.

While one case reports hypertriglyceridemia as the trigger of acute pancreatitis, which then causes DKA [11], in another case, DKA is suggested to be the cause of hypertriglyceridemia and acute pancreatitis [9].

Several neurological deficiencies have been associated with DKA, including cerebral edema with increased intracranial pressure resulting in a coma, partial and generalized seizures, and cerebrovascular occlusive disease resulting in motor and/or sensory dysfunction [12]. Neurological deterioration during an episode of DKA is usually assumed to be caused by cerebral edema, but hemorrhagic infarction is an infrequent cause. During an episode of DKA, increased inflammation increases the risk of vascular disruption, as well as hyperglycemia and acidosis-induced oxidative injury [13]. Acute renal involvement in DKA is probably affected by multiple factors, but it is most likely due to hypotension and hypovolemia [14]. Myers et al. [15] found an association between renal involvement and signs of cerebral injury with DKA.

To our knowledge, this is a very rare case involving the terrible triad and required immediate medical management for the control of DKA as well as hypertriglyceridemia.

## Conclusions

We report a rare DKA case with extremely high triglycerides complicated by acute pancreatitis, renal involvement, and a hemorrhagic infarct, which was managed with fluids and insulin. Early diagnosis of the terrible triad helps identify the complications, and proper management helps prevent its long-lasting adverse effects. However, further research into this triad is required to understand its pathophysiology and effects on the human body thoroughly.

## References

[1] Kitabchi AE, Nyenwe EA (2006). Hyperglycemic crises in diabetes mellitus: diabetic ketoacidosis and hyperglycemic hyperosmolar state. Endocrinol Metab Clin North Am.

[2] Culina S, Brezar V, Mallone R (2013). Insulin and type 1 diabetes: immune connections. Eur J Endocrinol.

[3] Loscalzo J, Fauci A, Kasper D, Hauser S, Longo D, Jameson JL (2022). Harrison's principles of internal medicine, 21st edition.

[4] Hahn SJ, Park JH, Lee JH, Lee JK, Kim KA (2010). Severe hypertriglyceridemia in diabetic ketoacidosis accompanied by acute pancreatitis: case report. J Korean Med Sci.

[5] Fortson MR, Freedman SN, Webster PD 3rd (1995). Clinical assessment of hyperlipidemic pancreatitis. Am J Gastroenterol.

[6] Chaurasiya OS, Kumar L, Sethi RS (2013). An infant with milky blood : an unusual but treatable case of familial hyperlipidemia. Indian J Clin Biochem.

[7] Furuya T, Komatsu M, Takahashi K (2002). Plasma exchange for hypertriglyceridemic acute necrotizing pancreatitis: report of two cases. Ther Apher.

[8] Denecker N, Decochez K (2013). Poorly controlled type 2 diabetes complicated by an episode of severe hypertriglyceridaemia-induced pancreatitis. BMJ Case Rep.

[9] Shaikh BH, Sohaib M, Alshantti R, Barrera F, Faridi FS, Murvelashvili N (2018). Diabetic ketoacidosis and the domino effect. Am J Case Rep.

[10] Joustra ML, Raidt JJ, Droog F, Veneman TF (2020). Diabetic ketoacidosis, hypertriglyceridemia and abdominal pain due to acute pancreatitis complicated by non-immune haemolytic anaemia. Eur J Case Rep Intern Med.

[11] Donelli D, Morini L, Trenti C, Santi R, Arioli D, Negri EA (2018). Plasma exchange for the treatment of transient extreme hypertriglyceridemia associated with diabetic ketoacidosis and acute pancreatitis. Eur J Case Rep Intern Med.

[12] Atluru VL (1986). Spontaneous intracerebral hematomas in juvenile diabetic ketoacidosis. Pediatr Neurol.

[13] Foster JR, Morrison G, Fraser DD (2011). Diabetic ketoacidosis-associated stroke in children and youth. Stroke Res Treat.

[14] Murdoch IA, Pryor D, Haycock GB, Cameron SJ (1993). Acute renal failure complicating diabetic ketoacidosis. Acta Paediatr.

[15] Myers SR, Glaser NS, Trainor JL (2020). Frequency and risk factors of acute kidney injury during diabetic ketoacidosis in children and association with neurocognitive outcomes. JAMA Netw Open.

